# An Unusual Paraganglioma of the Left Vagus Nerve: A Surgical Conundrum

**DOI:** 10.7759/cureus.84219

**Published:** 2025-05-16

**Authors:** Antonio Castillo Magaña, Elias Gallardo-Navarro, Catalina Gonzalez Aguirre, Grece Daniela Salinas-García

**Affiliations:** 1 Head and Neck Surgery, Hospital Español, Mexico City, MEX; 2 General Surgery, Hospital Español, Mexico City, MEX; 3 Oncologic Surgery, Hospital Español, Mexico City, MEX

**Keywords:** cervical paraganglioma vagal, extra-adrenal paraganglioma, glomus vagal tumour, head and neck tumors, surgical treatment

## Abstract

Vagus nerve paragangliomas (PGL) are tumors derived from neural crest cells of very low incidence, and cervical PGL of the vagus nerves (VPGLs) are tumors, of which the nature and location make them extremely rare, representing only 0.012% of cervical tumors, with an incidence of 1:30,000-1:200,000. A 76-year-old woman presented with a rapid growth in volume in the left laterocervical region, accompanied by pain for four months. She denied voice changes, difficulty feeding, facial nerve paralysis, headache, hypertension, hearing loss, excessive sweating, tinnitus, and tremors. Catecholamines were requested in blood or urine, which were negative. A computed tomography angiography of the head and neck was performed, where a tumor was identified whose location and characteristics were diagnosed as a PGL of the left vagus nerve; no biopsy was performed. Head and neck surgeons should be aware of large vagal PGL involving nerve or vascular structures, as it is currently difficult to resect the tumor without sacrificing the vagus nerve, and postponing surgery may also increase patient morbidity.

## Introduction

The cervical paragangliomas (PGL) of the vagus nerve (VPGLs) are tumors that occur in the head and neck very infrequently, making their diagnosis difficult. They represent about 0.012% of cervical tumors, with an incidence of 1:30,000-1:200,000, with a higher incidence in the female sex. These tumors can be divided into sympathetic and parasympathetic. The natural behavior of this type of tumor is benign and does not produce catecholamines [1,2]. The somatic motor and sensory, preganglionic neurons, and supporting cells of the cranial nerve ganglia embryologically originate from cells of the neural crest, the path of the vagus nerve descends vertically through the neck along with the carotid bundle that includes the internal jugular vein, vagus nerve and carotid artery then enter the mediastinum located on the left and right side, and this nerve is the communication pathway between the central nervous system and the viscera [3,4].

Rodríguez-Bustabad et al. suggest that PGL be named according to their anatomical location; thus, we could observe carotid body paraganglioma, jugulotympanic paraganglioma, and vagal paraganglioma in the cervical area [2]. The appearance of these tumors can occur at any age, with a higher prevalence between the third and sixth decades of life. The usual location is in the adrenal gland. Another region where they are found is in the abdomen and pelvis and, less frequently, in the region of the head and neck; in this region, characteristics such as non-painful, mobile, and slow-growing should not be confused with other types of lesions [5,6].

These tumors can be sporadic or familial, and it is estimated that the incidence of secretory tumors with symptoms such as paroxysmal hypertension, headache, palpitations, and sweating is less than 1-3% of all head and neck PGL. On the other hand, 19% manifest malignant behavior, destroying adjacent structures, and 10-20% metastasize to regional lymph nodes or lungs or cranial brain structures [6].

The presentation of these tumors can be unilateral, bilateral, or multicentric; an important risk factor when these multicentric tumors occur is to rule out a mutation in the tumor suppressor gene hereditary paraganglioma syndrome type 1 (PGL-1) on chromosome 11. They are usually unilateral and solitary in more than 90%, and the rest can be multiple and bilateral. When they have a family history, 40% of these cases present a mutation in the germline of one of the 20 predisposing genes currently found [7,8]. One of these genes, those encoding the enzyme succinate dehydrogenase (SDH), are the most frequently implicated, and, when identified, have a higher degree of malignancy [7,9]. When PGL occur in familial forms, these tumors may occur as solitary tumors or be accompanied by other tumors that occur in syndromes such as multiple endocrine neoplasia, Von Hippel-Lindau syndrome, and neurofibromatosis type 1 [9].

The genes involved in the disease are located at three loci: SDHD (11q23), SDHC (1q21), and SDHB (1p36, 1p35), and one-third of sporadic PGL also have SDH mutations identified in the germline [7,9]. In general, PGL are vascular tumors arising from the principal cells of the extra-suprarenal paraganglionic tissue of the autonomic nervous system. Vagal PGL occur along the vagus nerve, usually located in the rostral portion of the nerve in the vicinity of the ganglioglioma [3,10,11].

## Case presentation

**Figure 1:**
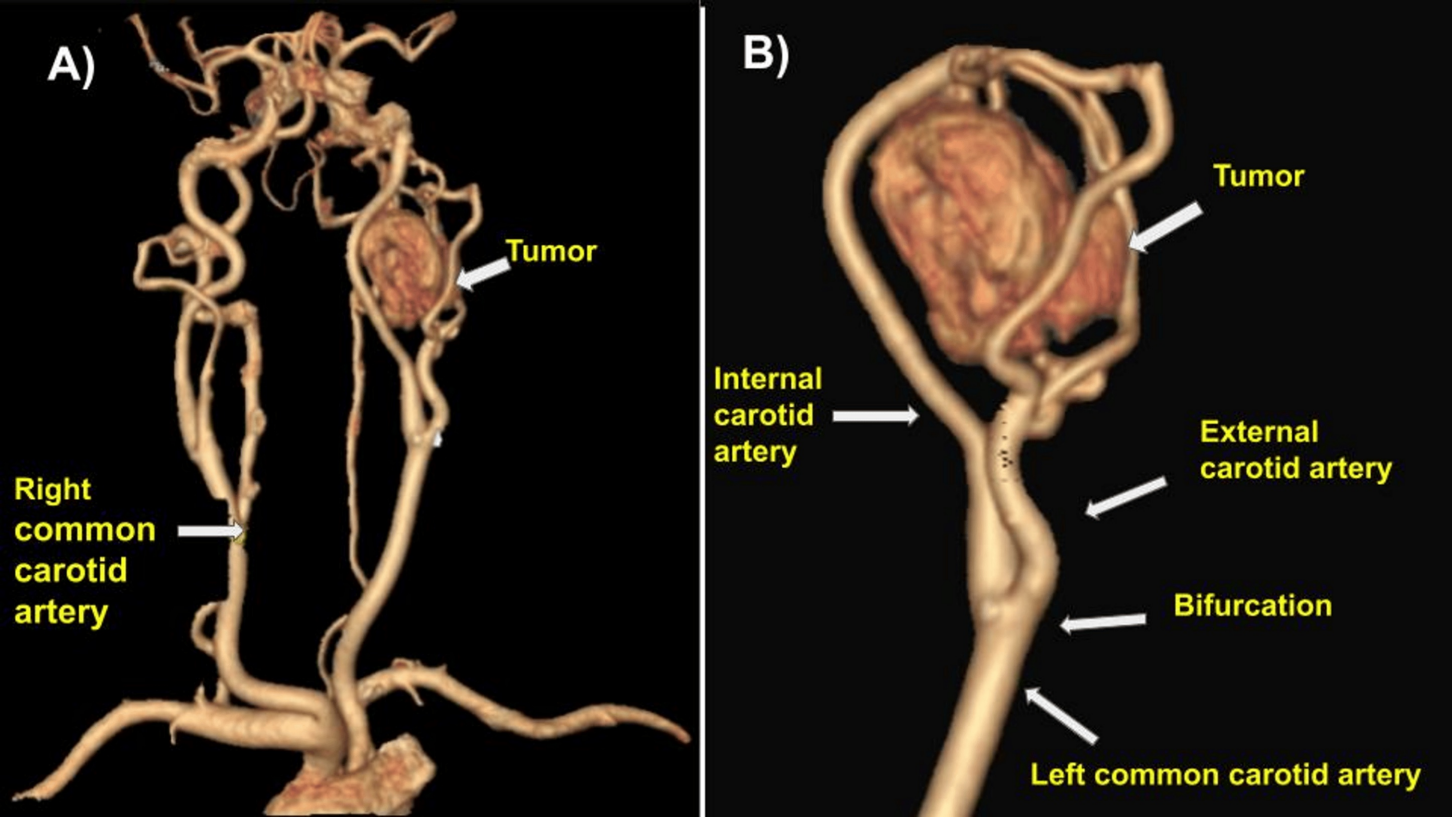
Computed angiotomography (A) The tumor is seen 2 cm above the left carotid bifurcation. (B) This tumor dislocates the anterior internal and external carotid arteries. Image credit: Authors

A 76-year-old Latin American woman, originally from Mexico City, Mexico, with no surgical history or significant comorbidities of the current disease, denies smoking and alcoholism and has a negative family history. For the last four months, the patient has been complaining of pain and increased volume in the left latero-cervical region, so she went to see her family doctor, who gave her analgesic treatment. When there was no improvement in the pain and persistence of the increased volume in the same region, she went for medical consultation with the head and neck surgery specialist, where a multislice computed angiotomography of the neck was requested. A solid, oval tumor was identified 2 cm above the left carotid bifurcation, at the height of the vertebral body C2, with density in the simple phase of 31.8 HU, which presents an avid peripheral enhancement with the contrast medium during the arterial phase, showing a hypodense central area (Figure 1).

**Figure 2:**
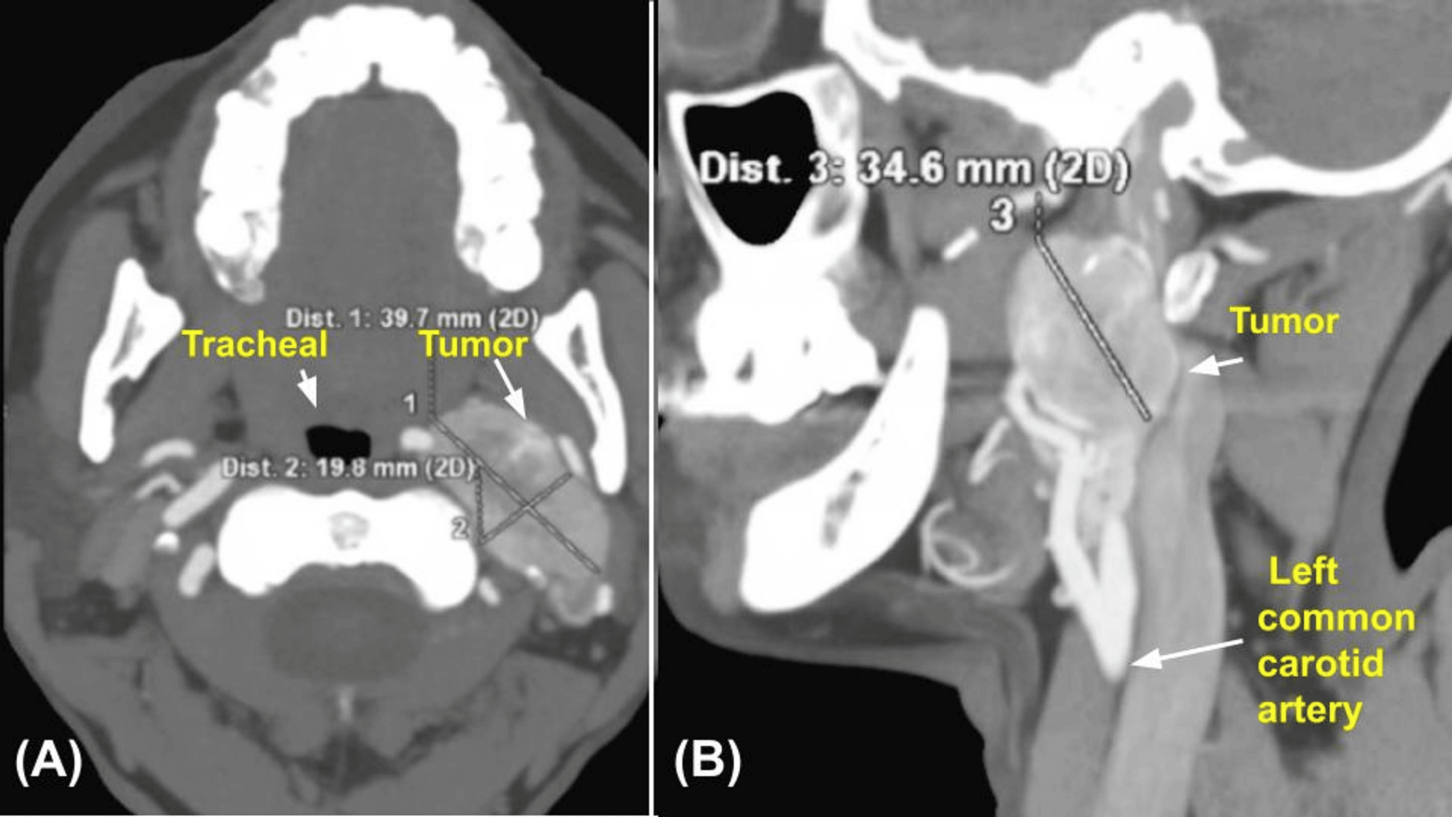
Computed angiotomography (A) Axial cut: the tumor is observed, limited that displaces the trachea. (B) Sagittal cut: increased calibre of the external and internal carotid is observed.

**Figure 3:**
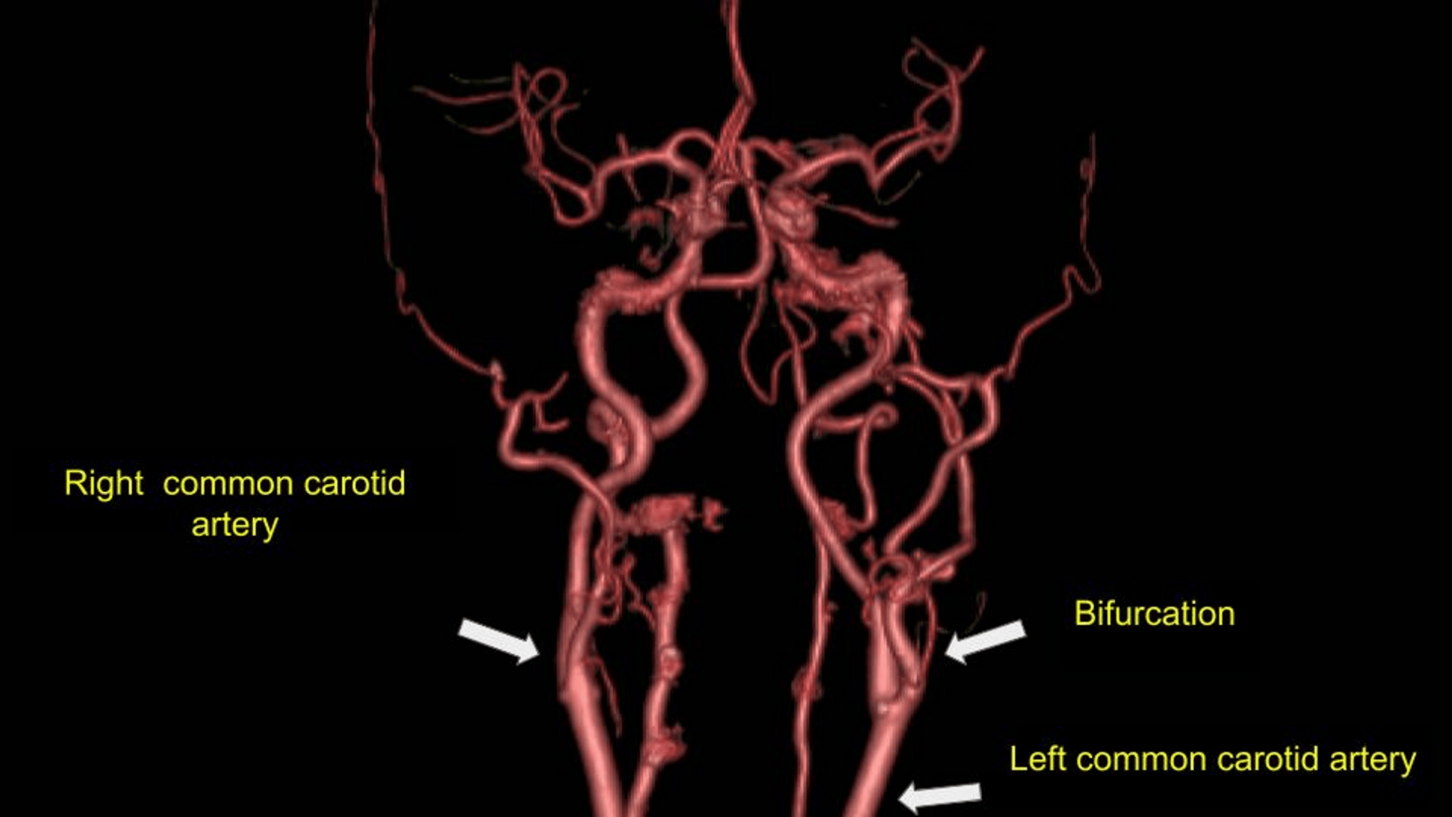
CT: 512 slices were used in single phase, and after administration of non-ionic intravenous contrast medium (vol. 50 mL), there is no evidence of solid hypervascular lesions in the right carotid bifurcation Image credit: Authors

This tumor displaced vascular structures, while it displaced posteriorly the ipsilateral internal jugular vein, measuring approximately 3.46 x 1.98 x 3.97 cm in the longitudinal, anteroposterior, and transverse axes, respectively (Figure 2). There is no evidence of solid hypervascular lesions in the carotid bifurcation on the right side (Figure 3); the suggestive diagnosis of the study is a PGL of the left vagus nerve. Physical examination revealed increased volume in the left anterolateral cervical region, mobile, soft, no color changes in the skin region, and negative cervical nodes. On diagnostic questioning, she denied voice changes, difficulty feeding, facial nerve palsy, headache, high blood pressure, hearing loss, excessive sweating, tinnitus, and tremors. Laboratories were requested and found to be within normal parameters; no tumor markers or catecholamines were requested in blood or urine.

**Figure 4:**
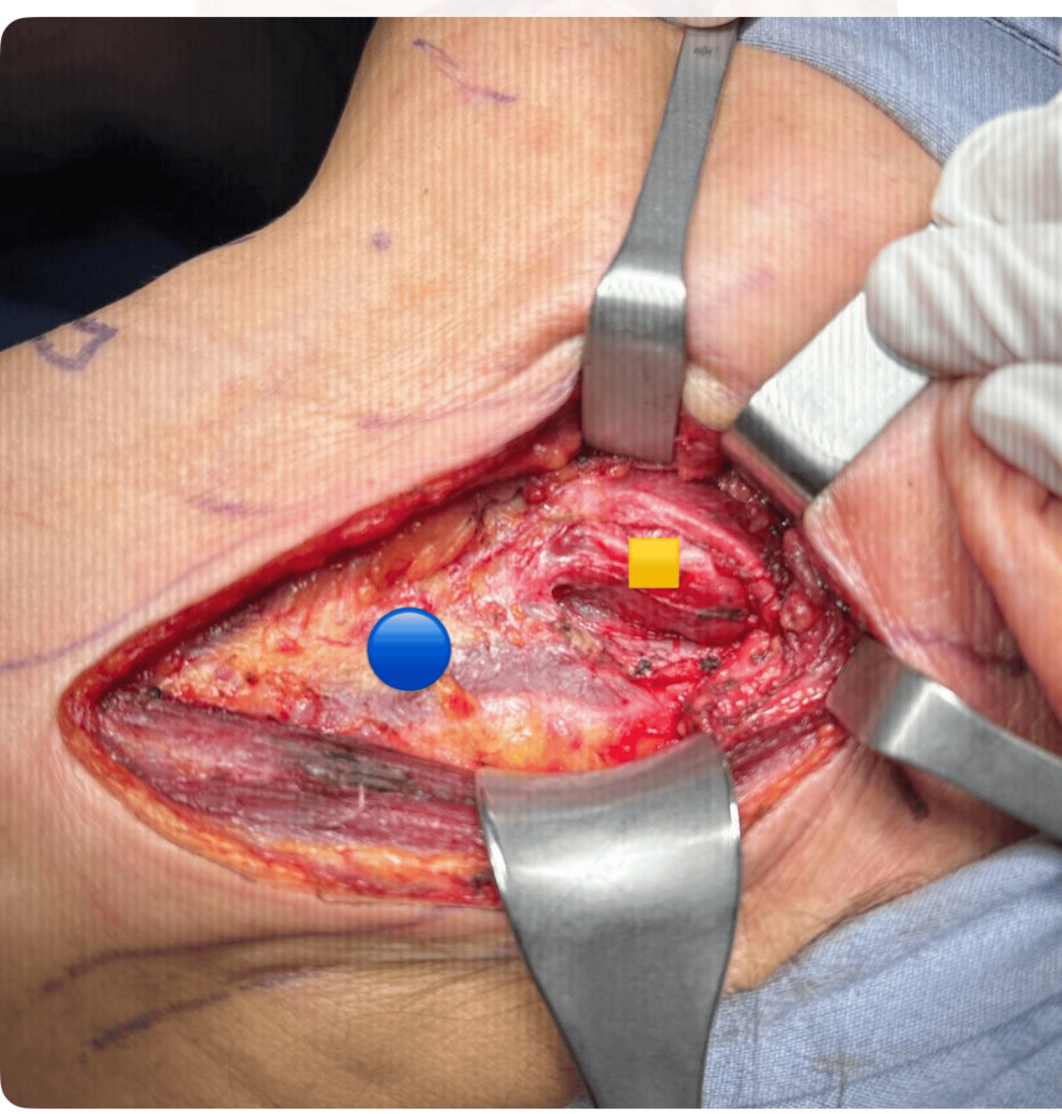
Intraoperative image Blue circle: deep cervical prevertebral fascia; orange square: left vagus nerve paranglioma

The tumor was resected under general anesthesia; during surgery, vital signs remained within normal parameters. There was no need to add beta-blocker drugs, and neuromonitoring of the vagal, hypoglossal, and spinal nerves was in place at all times. A left laterocervical longitudinal incision was made, extending to the mastoid process, and the tumor was dissected, which had its origin in the left vagus nerve. The lower edge of the posterior belly of the digastric muscle was incised with electrocautery, and then traction was performed towards the cephalic. The common carotid sheath was identified, and the sheath was incised (Figure 4). The carotid artery with electrocautery and an ovoid tumor of approximately 5 x 4 cm, located 3 cm above the carotid bifurcation, was observed.

**Figure 5:**
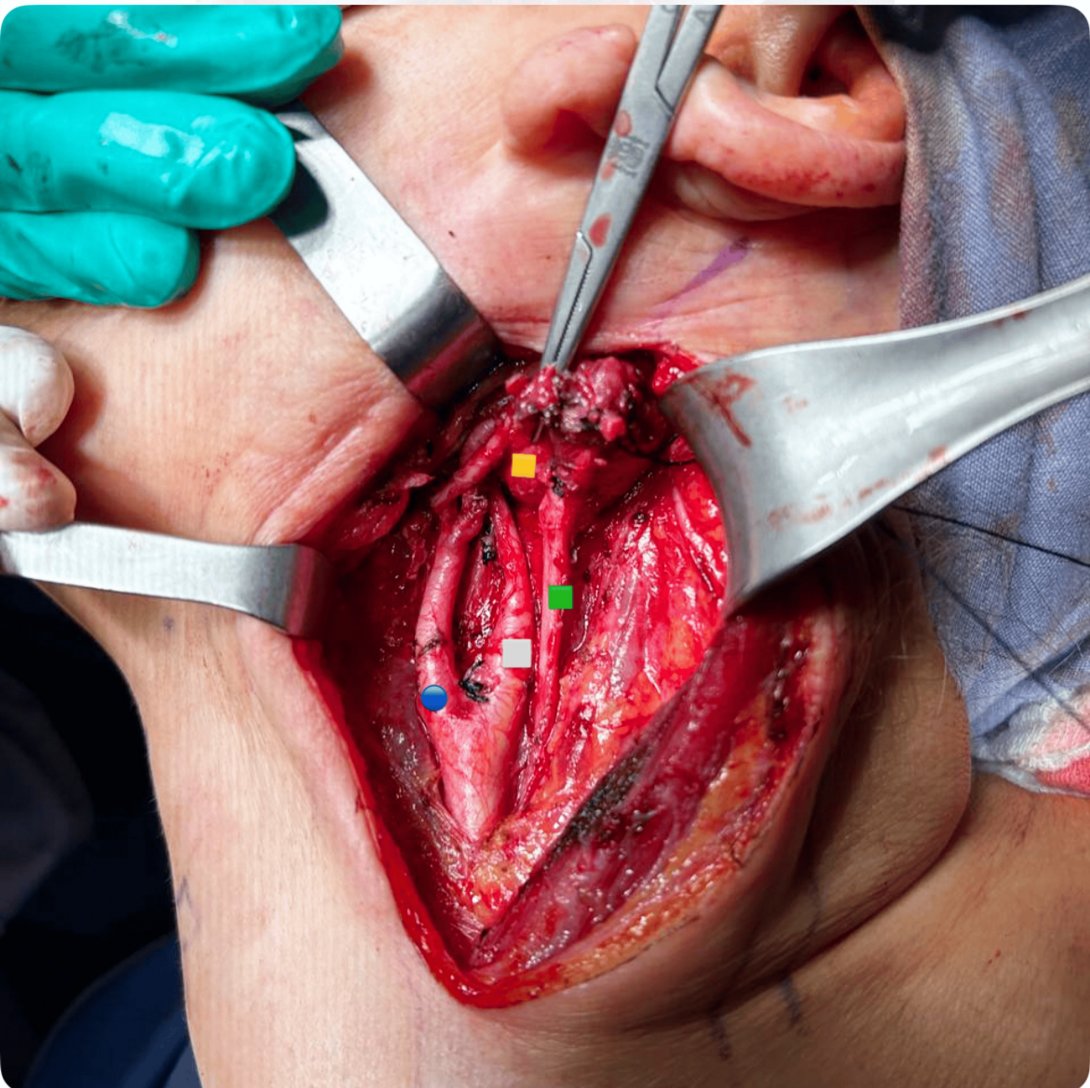
Intraoperative image Blue circle: left internal carotid artery; white square: left internal carotid artery; green square: left vagus nerve; orange square: paranglioma of the vagus nerve.

**Figure 6:**
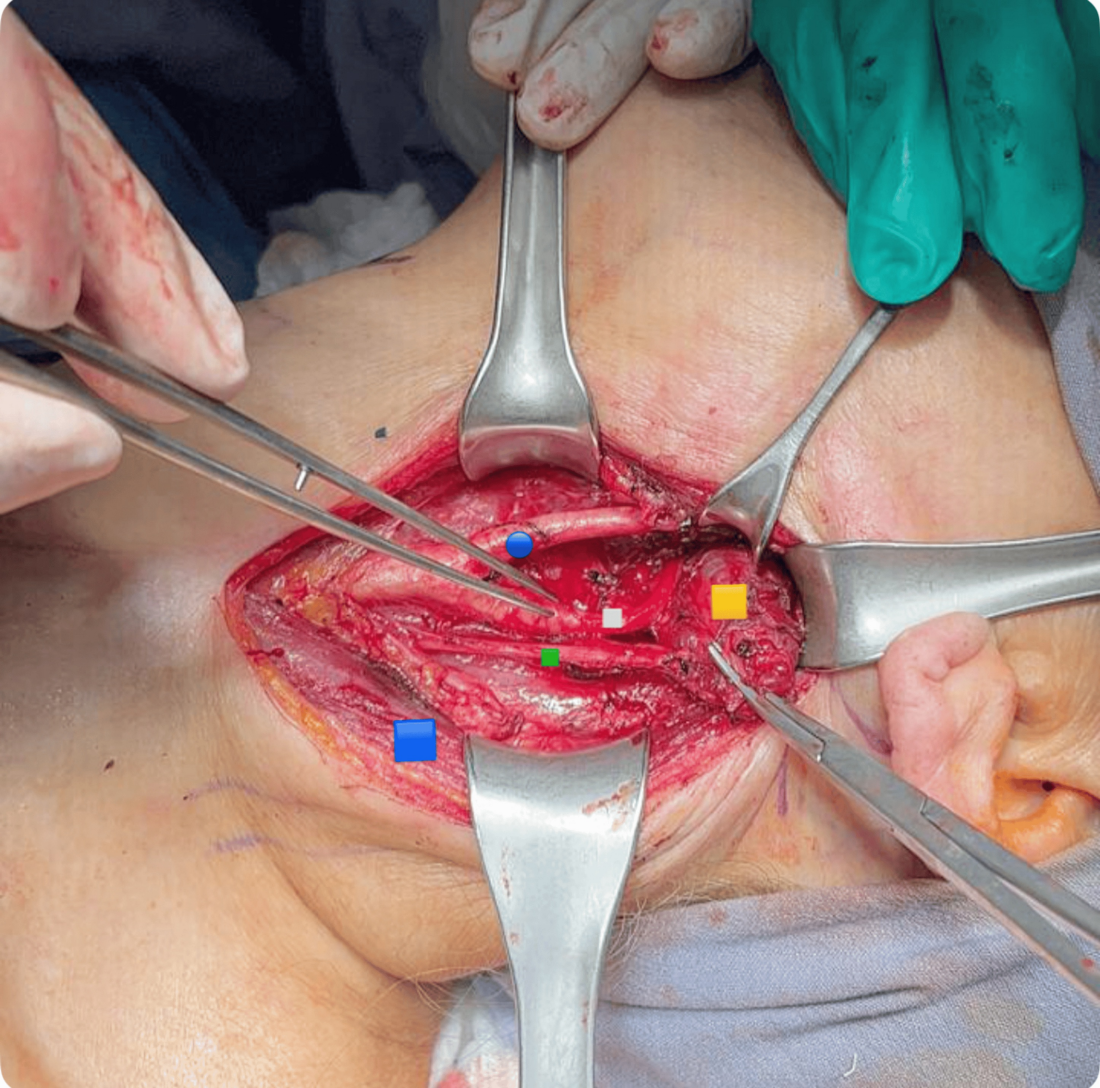
Intraoperative image Blue circle: left internal carotid artery; white square: left internal carotid artery; green square: left vagus nerve; orange square: vagus nerve paranglioma; blue square: sternocleidomastoid muscle

Lateralization of the vagus nerve, internal jugular, and carotid on the left side, together with its bifurcation, internal and external carotid. Dissection of the edges of the tumor is performed with advanced and monopolar energy under neuromonitoring of the left vagus nerve (Figures 5-6). Dependence of the tumor on the vagus nerve and frank involvement of the left hypoglossal nerve was observed, making its resection impossible without injuring the aforementioned nerves. It was decided to resect the tumor, the edges were released in its entirety with a section of the left vagus nerve and left hypoglossal nerve, and the spinal nerve was observed intact. The piece was removed and sent for histopathological study with a report of PGL of the left vagus nerve, and the immunohistochemistry panel was positive for the markers Bcl-2, Ki-67, and Bax.

Hemostasis was performed and verified. The muscular plane was approached with 3-0 Vycryl. The skin was approached with monocryl 4-0s, and the wound was covered. The patient has an adequate postoperative period with difficulty in swallowing soft foods, due to the compromise of the hypoglossal nerve and hoarseness, so she is discharged from the hospital 72 hours after surgery, with follow-up in swallowing and phonation rehabilitation therapy, with no other added morbidity at 15 days, one month, and two months of follow-up.

## Discussion

We present an uncommon case, as far as the presentation of the symptomatology is concerned. Since most of the clinical manifestations of the patient are related to the origin of the tumor, the symptomatology depends mainly on the location. For example, if it is located within the parapharyngeal space, typically, these masses tend to be soft and symptomatic until they reach a large size that can compress adjacent structures, such as blood vessels or nerves, causing deficits of the nerves involved and compression of the airways, such as the trachea. Other clinical presentations may include Horner syndrome, dysphagia, syncope, dysphonia, pain, and fullness of the pharynx. Our patient, whose only risk factor was being a woman, did not present symptoms associated with the release of catecholamines, nor the symptoms previously discussed [4,11]. Other authors consider that the ganglion of origin of the vagal glomus may determine the presentation of the symptomatology and describe the growth pattern of the tumor such that those located in the nodal ganglion regularly remain in the cervical region, while those located in the median ganglion approach the base of the skull and those in the superior ganglion may develop intracranially [10,11].

Cranial nerve deficits are also common at presentation, with the vagus nerve and hypoglossal nerves experiencing deficits of around 28-37% and 17-21%, respectively. Other less commonly involved cranial nerves include the spinal accessory, glossopharyngeal, and facial nerves [12,13]. One of the characteristics of vagal PGLis that they displace adjacent structures, such as the anterior carotid artery, while tumors located in the carotid body displace it later and may develop towards the jugular foramen, simulating a jugular PGL, and may also extend towards carotid bifurcation in the posterior or inferior trench [13-15].

It is a diagnostic challenge when a cervical mass is addressed, due to the importance of the structures that are located in this region, thus the delimitation of whether a tumor is in a deep location or is located in superficial tissues. This facilitates the diagnostic approach, so the use of computed tomography, magnetic resonance imaging (MRI), and angiography is recommended, either to try to diagnose a particular pathology or tumor or to plan some surgical treatment based on its size. Computed tomography helps delimit the invasion of tumours, mainly to the bone parts; magnetic resonance imaging provides greater clarity of infiltration or surrounding adjacent soft tissues; and angiography helps narrow the vascularity or involvement of large vessels if there are any [15,16].

Genetic testing is recommended in patients with any of the following risk factors: age >50 years, family history, extra-adrenal tumors, multiple or metastatic tumors, and elevated levels of dopamine and methoxytyramine. Hence, it was not performed in our patient since the only associated factor was age, so it was not considered necessary in this case [16].

Another method of evaluation used in patients with a history of vagal PGL of familial origin is the use of pentetreotide scintigraphy of indium. This contrast medium is followed by a series of radiographic images, allowing small tumours to be observed, which, when diagnosed in a timely manner, allows surgical resection without the need to sever any nerve [11,14,15]. Morphologically, PGL are the same as glomus cells (type I) of the carotid body; these cells are located inside the nerve or next to the vagus nerve; the two types of cells share the characteristic “zellballen” pattern, which is a reticular network of interlaced neuroepithelial sheets of variable thickness, separated by an abundant microvascular bed; and, microscopically, this pattern takes the morphology of rounded or oval elongated cell groups [15,16].

Immunohistochemistry markers for the diagnosis of neuroendocrine tumors are part of the diagnosis of PGL, and the markers included in the immunohistochemistry panel are Bcl-2, Ki-67, Bax, and pituitary adenylate cyclase activating peptide (PACAP), somatostatin, vasoactive intestinal peptide (VIP), and calcitonin gene-related peptide (CGRP), to increase diagnostic specificity. Some of these markers have prognostic value [17].

There are three possible extensions of the tumor, which must be known to address this tumor surgically. Sniezek et al. [12] described this based on the extension and size of the tumor in the region where it spreads, since the tumor may be confined in the cervical region or parapharyngeal space, or from the cervical region, and can spread to the base of the skull to the jugular foramen and cause displacement of the carotid artery. Knowing the tumor extension and its behavior, as a surgeon, is the best way to approach it [17,18].

A recommended technique within the resection of these tumors is the careful periadventitial dissection of vascular structures such as the carotid artery, in order to avoid damage to the arterial wall. Sometimes, the ligation and resection of the external carotid artery improves the visual field, decreases the risk of bleeding, facilitates the resection of larger vagal PGL, and reduces intraoperative bleeding. Surgical resection is the treatment of choice for vagal PGL. Since resection can prevent nerve involvement other than the vagus nerve, such as the hypoglossal and glossopharyngeal nerve, the malignant invasion that can complicate your resection is different for PGL types that are carotid or tympanic. Thus, timely recognition is the best way to address them to avoid unnecessary resections. Resection of vagal PGL invariably involves sacrificing the vagus nerve, as they usually come when they produce symptoms of a large volume and are therefore rarely treated at an early stage when vagal PGL are very small and do not sacrifice the nerve [17-19]. 

Surgical excision is the primary treatment modality for head and neck vagal PGL; currently, the relative effectiveness of surgery versus radiotherapy remains controversial. While radiotherapy can significantly slow tumor growth and provide palliation in selected patients, in most cases, tumor size does not decrease or change appreciably with radiation, and the presence of histologically viable tumor cells (chief cells) after radiotherapy has been demonstrated. Conventionally, nonsurgical treatment for malignant unresectable VPGLs includes radionuclide therapy and the administration of alkylating agents or tyrosine kinase inhibitors [19,20].

## Conclusions

Head and neck surgeons must take into account large vagal PGL involving nervous or vascular structures; since it is currently difficult to remove the tumor without sacrificing the vagus nerve, being a very rare tumor, the diagnostic suspicion is based on the discard or possibly by family history. However, when symptoms are present, they are now large tumors, so the morbidity and mortality of the patient and complications increase. Vagus nerve PGL are tumors with the greatest postoperative neurological involvement, they are also commonly more aggressive, and, currently, no preoperative criteria predictive of malignancy have been identified. The risk of malignancy is difficult to estimate because the only recognized criterion is the cervical lymph node or distant metastases. Surgical resection followed by radiotherapy is the most common treatment described for malignant VPGLs, even if its rare occurrence is not clear. In our case, there was no need to resect the cervical nodes, and she is currently free of disease.
